# Vertical Jump Height Estimation Using Low-Sampling IMU in Countermovement Jumps: A Feasible Alternative to Motion Capture and Force Platforms

**DOI:** 10.3390/s24247877

**Published:** 2024-12-10

**Authors:** Giacomo Villa, Alessandro Bonfiglio, Manuela Galli, Veronica Cimolin

**Affiliations:** 1 Euleria srl Società Benefit, Via delle Zigherane, 4/A, 38068 Rovereto, Italy; giacomo1.villa@polimi.it (G.V.); alessandro.bonfiglio@unitn.it (A.B.); 2 Department of Electronics, Information and Bioengineering (DEIB), Politecnico di Milano, 20133 Milano, Italy; manuela.galli@polimi.it; 3 Department of Information Engineering and Computer Science (DISI), University of Trento, 38121 Trento, Italy; 4 Energy Efficient Embedded Digital Architectures (E3DA), Fondazione Bruno Kessler, 38121 Trento, Italy; 5 Istituto Auxologico Italiano, IRCCS, S. Giuseppe Hospital, Oggebbio, 28824 Piancavallo, Italy

**Keywords:** countermovement jump, vertical jump height, inertial measurement unit, motion capture systems, force platforms, double integration

## Abstract

Vertical jump height from a countermovement jump is a widespread metric to assess the lower limb functionality. Motion capture systems and force platforms are considered gold standards to estimate vertical jump height; however, their use in ecological settings is limited. This study aimed to evaluate the feasibility of low-sampling-rate inertial measurement units as an alternative to the gold standard systems. The validity of three computational methods for IMU-based data—numerical double integration, takeoff velocity, and flight time—was assessed using data from 18 healthy participants who performed five double-leg and ten single-leg countermovement jumps. The data were simultaneously collected from a motion capture system, two force platforms, and an IMU positioned at the L5 level. The comparisons revealed that the numerical double integration method exhibited the highest correlation (0.87) and the lowest bias (2.5 cm) compared to the gold standards and excellent reliability (0.88). Although the takeoff velocity and flight time methods demonstrated comparable performances for double-leg jumps, their accuracy in single-leg jumps was reduced. Overall, the low-sampling-rate IMU with the numerical double integration method seems to be a reliable and feasible alternative for field-based countermovement jump assessment, warranting future investigation across diverse populations and jump modalities.

## 1. Introduction

Vertical jumps (VJs) are a commonly assessed motor task in sport and clinical settings, providing valuable insights into lower limb function [1]. As a reliable benchmark for performance assessment, VJs are widely used during training to assess athletic ability [2], identify injury risks, and guide rehabilitation programs [3]. Among the various types of VJs, countermovement jumps (CMJs) are particularly widespread due to their widely proven reliability and validity in estimating the explosive power of the lower limbs [4]. The simplicity of the testing protocols, the repeatability, and the higher ecological validity have made CMJs an ideal tool for the assessment and comparison of lower limb performance across different sports [5,6,7,8,9], age groups [10,11,12], and sexes [13]. Furthermore, inter-limb asymmetries in CMJ metrics have been shown to correlate with injury rates, increasing research interest in this assessment as a valuable tool for injury prevention [14].

CMJs are characterized by an unloading downward phase, a breaking phase, a propulsive upward phase, a flight phase, and a final landing phase [4]. Although several metrics can be calculated for each jump phase, performance-related variables, such as jump height and the modified reactive strength index, have consistently demonstrated the highest reliability and applicability across diverse groups [4]. In particular, the most effective and investigated measure of CMJ performance is vertical jump height (VJH) [4,15,16].

To estimate VJH, researchers have historically relied on motion capture systems (MCap) and force platforms (FPs), which are widely acknowledged as the gold standard tools in the biomechanics field [17,18,19]. Despite their accuracy, these systems require a laboratory environment, restricting their applicability in ecological settings [20]. Although several portable FPs have been developed to enhance practicality, they often remain less cost-effective and less adaptable to irregular terrains compared to alternative devices, such as linear transducers, photoelectric cells, camera-based smartphone applications, or inertial measurement units (IMUs) [19,21,22,23].

IMUs combine accelerometers, gyroscopes, and magnetometers to collect raw acceleration and angular velocity data [22]. By leveraging sensor fusion algorithms, IMUs enable the extraction of kinematic information, facilitating the tracking of three-dimensional body movements [24]. Their compact size, versatility, accessibility, and low power consumption make IMUs an increasingly preferred tool for research applications in diverse settings, ranging from athletic performance to rehabilitation [22,24,25,26]. Over the last decade, IMU-based systems have been used for both rehabilitation and sport performance assessment purposes, with applications in various clinical and sportive contexts, including CMJ assessment [22,27,28,29,30].

Nevertheless, ensuring the accuracy and precision of these devices is essential to provide practitioners with accurate and reliable information [25]. High-sampling-rate IMUs (>100 Hz) have been validated for VJH estimation by comparing them with the gold standard reference systems [25]. However, in practical applications, low-sampling-rate IMUs are preferred because of their reduced power consumption, which extends battery life and allows data recording over longer periods [31]. Moreover, the low sampling rate also reduces the amount of data collected, simplifying data processing and storage, which can be critical in field-based applications or when multiple sensors are used simultaneously. Despite these practical advantages, lower sampling rates may reduce the accuracy of calculated metrics, such as VJH, due to the potential under-sampling of the jump’s dynamic phases [31]. Therefore, it is crucial to evaluate whether low-sampling-rate IMUs can still provide accurate and reliable estimates of VJH compared to high-sampling-rate devices and gold standard systems.

Several studies have shown that the choice of the appropriate calculation method according to the VJH definition chosen is crucial when validating technological tools [16]. In the present study, VJH is defined as the difference between the maximum height reached by the center of mass (COM) during the jump (H_max_) and the standing height (H_0_) [16]. Therefore, VJH calculated from the MCap system consists in the tracking of the vertical displacement of the COM, which is approximated from the positions of the markers placed on the subject’s body [32,33,34]. In contrast, FPs do not directly provide vertical displacement, necessitating the use of specific calculation methods to estimate VJH from vertical force data. The numerical double integration (NDI) method has been proven to be one of the most reliable and accurate approaches to quantifying VJH when calculated as the difference between H_max_ and H_0_ with a dual FP setup [16,35,36]. In addition, it has been proven that the NDI method provides VJH estimates with the most negligible difference compared to MCap [16,35,36].

While numerous studies have evaluated the validity of IMU sensors against the gold standard systems, there is still a lack of a standardized method for calculating VJH from IMU-derived data [25]. When using IMU data, VJH can be computed using three main calculation methods: (a) NDI, (b) the takeoff velocity (TOV) method, and the (c) flight time (FT) method [20]. The NDI method involves double integration of vertical acceleration, assuming zero initial acceleration, and the calculation of VJH as the maximum vertical displacement. The TOV method assumes the subject is only influenced by gravity during the jump, treating the body as a particle under constant acceleration (*g*), with takeoff velocity determined by integrating filtered acceleration data before takeoff (TO). The FT method relates VJH to FT, which is defined as the interval between TO and landing (LA) [37].

Several studies have validated these methods for calculating VJH with high-sampling-rate IMU sensors. However, to date, no studies have investigated the feasibility of low-sampling-rate IMUs for this purpose [16,25]. In this study, it was hypothesized that low-sampling-rate IMUs could serve as a feasible and compact alternative to the gold standard systems for estimating VJH in CMJs. This hypothesis was tested by using a single low-sampling-rate IMU to calculate VJH using the NDI, TOV, and FT methods. Additionally, the most accurate method for estimating VJH from IMU data was identified. Finally, a test–retest paradigm was implemented to determine the reliability of such method.

## 2. Materials and Methods

The study was conducted at the “Luigi Divieti” Posture and Movement Analysis Laboratory (Department of Electronics, Information and Bioengineering (DEIB), Politecnico di Milano, Milano, Italy) in accordance with the postulates of the Declaration of Helsinki and with the approval of the Ethics Committee of the Politecnico di Milano (Protoc. No. 22/2021, 14 June 2021).

### 2.1. Participants

Eighteen healthy participants (age: 24.6 ± 1.9 years, height: 169.4 ± 7.5 cm, and body mass: 56.2 ± 10.4 kg) voluntarily participated in the study and provided their written informed consent for the use of their data. The participants were included according to the following criteria: (i) age over 18 years; (ii) BMI between 18.5 kg/m^2^ and 24.9 kg/m^2^; (iii) absence of neurological or musculoskeletal impairments, and (iv) absence of injury or surgery within the previous six months.

### 2.2. Equipment

Data were collected simultaneously from the following three systems:

- An MCap system composed of eight infrared cameras (SMART DX 100, BTS-Bioengineering, Milan, Italy) with an accuracy of <0.2 mm over a 2 × 2 × 2 m volume and a sampling frequency of 100 Hz.
- Two FPs (AMTI, USA Inc., Watertown, MA, USA) measuring 464 × 508 × 82.5 mm with an accuracy of ±0.1% of the applied load and a sampling frequency of 200 Hz.
- An IMU (XSens DOT, Xsens Technologies B.V, Enschede, the Netherlands) with Bluetooth Low Energy (BLE) wireless connectivity and 60 Hz sampling frequency. It provides triaxial accelerations ([m/s^2^], ±16 g full scale), triaxial angular velocities ([°/s], ±2000 °/s full scale), and magnetic field measurements within the sensor’s fixed frame.

To synchronize data across all the systems, the signals were resampled to the lowest common sampling rate (i.e., IMU sampling frequency, 60 Hz).

### 2.3. Procedure

The participants were informed of the purpose of the study and their height and body mass were recorded prior to testing. Before the experimental session, the participants completed a standard warm-up consisting of 4 min of jogging on a treadmill at a self-selected pace, followed by 3 min of submaximal vertical jumps to familiarize themselves with the testing environment.

Subsequently, the participants were instrumented with three passive spherical markers placed on the sacrum and bilaterally over the anterior superior iliac spine. The IMU was mounted at the level of L5, identified by palpation as the midpoint between the posterior superior iliac spines. To minimize the effects of skin motion artifacts during jumps, the IMU was secured with a non-invasive elastic band. The participants were briefed on the proper execution of the CMJ, which involved an initial downward movement followed by a rapid upward jump without arm swing, ensuring minimal posture change to reduce potential effects on VJH. The depth of the countermovement was not controlled. The participants were then instructed to place each foot on an FP, and 5-s static data were recorded from the three systems prior to CMJ execution.

Each participant performed double-leg CMJs (DL-CMJs), right-leg CMJs (R-CMJs), and left-leg CMJs (L-CMJs), with at least 30 s of rest between each jump and 3 min of rest between each jump category, until 5 correct jumps were performed for each task. Therefore, motion data for a total of 90 jumps from the MCap, IMU, and FP for each jump modality were collected.

### 2.4. Data Processing

The motion data were analyzed using MATLAB (v.2023a, MathWorks, Natick, MA, USA) through customized algorithms. The VJH was computed for each system using a system-specific calculation method.

For the MCap data, the vertical trajectories of the three passive markers were averaged to approximate the vertical displacement of the pelvis COM [34]. The VJH was calculated as:

VJH_MCap_ = H_max_ − H_0_, (1)

where $H_{max}$ is the maximum height reached by the pelvis COM, calculated as the absolute maximum of the vertical trajectory, and $H_{0}$ is the height of the COM in the resting position obtained during the 5-s static recording.

Regarding the FP system, the vertical acceleration ($a_{vert}^{FP}$, [m/s^2^]) was derived, according to Newton’s second law, from the ratio between the vertical force signal ($F_{z}$, [N]) and the subject’s mass, with the gravitational acceleration (g, [m/s^2^]) subtracted:

(2) $$a_{vert}^{FP}=\frac{F_{z}}{\frac{F_{0z}}{g}}-g,$$

where $F_{z}$ is the vertical force signal obtained as the average of the vertical force from both the force platforms, and $F_{0z}$ is the vertical force during the 5-s static recording. The participant’s mass was derived by dividing $F_{0z}$ by g. A low-pass Butterworth filter (order = 4, cut-off frequency = 10 Hz) was applied to the $a_{vert}^{FP}$ and then double-integrated using the trapezoidal rule from the onset of movement until landing to obtain the vertical displacement signal, according to the NDI method [16,36]. The movement onset was defined as the point when $F_{z}$ dropped below 2.5% of $F_{0z}$ [38]. The VJH_FP_ was determined as the maximum value of the vertical displacement.

Lastly, the IMU’s vertical acceleration data were transformed from the local reference frame (*S*) to a fixed global reference frame (*G*) using quaternion-based rotation to address sensor potential misalignment during manual sensor placement on the participant’s trunk [39]. This transformation ensured that the acceleration data could be analyzed in a consistent global context, unaffected by variations in sensor placement or body orientation. In *S*, the *x*-axis was aligned with gravity pointing upwards, the *z*-axis was aligned with the forward direction relative to the participant’s sagittal plane, and the *y*-axis was orthogonal to the resulting XZ plane. In *G*, the *z*-axis was aligned with gravity, the *x*-axis lay within the global horizontal plane, corresponding to the participant’s forward direction when standing upright, and the *y*-axis was orthogonal to the ZX plane, completing the right-handed coordinate system. To describe the transformation required to rotate the acceleration vector from *S* to *G*, quaternion data—obtained as output from the IMU sensor, as in (3)—were used to derive a rotation matrix ($R_{G}^{S}$) for each time sample, defined as in (4):

(3) $$q={[q}_{w},q_{x},q_{y},q_{z}],$$

(4) $$R_{G}^{S}=\left[\begin{array}{ccc} 1-2(q_{y}^{2}+q_{z}^{2}) & 2(q_{x}q_{y}-q_{z}q_{w}) & 2(q_{x}q_{z}+q_{y}q_{w}\\ 2(q_{x}q_{y}+q_{z}q_{w} & 1-2(q_{x}^{2}+q_{z}^{2}) & {2(q}_{y}q_{z}-q_{x}q_{w})\\ 2(q_{x}q_{z}-q_{y}q_{w}) & 2(q_{y}q_{z}+q_{x}q_{w}) & 1-2(q_{x}^{2}+q_{y}^{2}) \end{array}\right],$$

The three-dimensional global acceleration vector was obtained by multiplying the acceleration vector in the local reference frame ($a^{S}$) by $R_{G}^{S}$. The absolute vertical acceleration $a_{vert}^{IMU}$ was extracted as the third component (*z*-axis) of the global acceleration vector and subtracting the contribution of g, as in (5):

(5) $$a_{vert}^{IMU}={\left[R_{G}^{S}\times {{(a}^{S})}^{T}\right]}_{z}^{T}-g,$$

The absolute vertical acceleration $a_{vert}^{IMU}$ was then filtered with a low-pass Butterworth filter (order = 4, cut-off frequency = 10 Hz) and used to compute VJH through three methods:

- The FT method is based on the identification of TO and LA events from $a_{vert}^{IMU}$. TO is defined as the instant when $a_{vert}^{IMU}$ crosses the zero-acceleration threshold, while LA is identified as the last observation of $a_{vert}^{IMU}$ less than 0. ${VJH}_{IMU}^{FT}$ is then obtained as in (6) [37]:
  (6) $${VJH}_{IMU}^{FT}=\frac{{\left(LA-TO\right)}^{2}\times g}{8}\;$$
- The TOV considers the subject as only affected by gravity during the jump and neglects the air resistance. ${VJH}_{IMU}^{TOV}$ is calculated from (7), where TOV is the takeoff velocity determined by the integration of $a_{vert}^{IMU}$ measured before the TO instant, as defined in the FT method [20]:
  (7) $${VJH}_{IMU}^{TOV}=\frac{{\left(TOV\right)}^{2}}{2g}\;$$
- The NDI consists in the double integration of the $a_{vert}^{IMU}$ via the trapezoidal rule to obtain the vertical displacement [20]. The ${VJH}_{IMU}^{NDI}$ was calculated as the maximum value of the vertical displacement [20].

**Figure 1:**
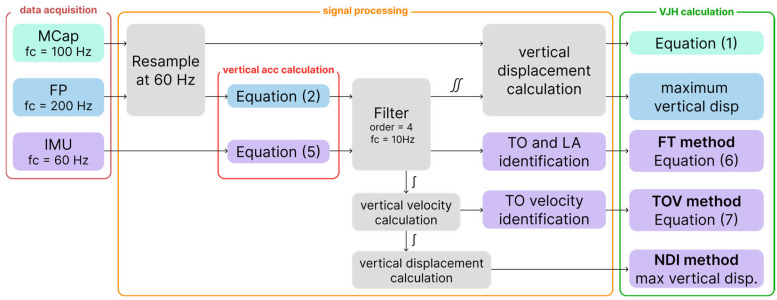
Main steps to obtain VJH from raw data of the three systems.

The main steps applied to the signals obtained from the different systems are summarized in Figure 1.

### 2.5. Statistical Analysis

The statistical analysis was performed using JASP (JASP Team 2023, Version 0.17.3) and MATLAB (v.2023a, MathWorks, Natick, MA, USA).

The normality of the VJH estimates from the different systems and IMU-based calculation methods was assessed using the Shapiro–Wilk test. The test indicated a non-normal distribution (*p* < 0.05), leading to the use of non-parametric statistical methods [40]. The descriptive statistics for each calculation method are presented as median and interquartile range (IQR).

The agreement between the two gold standard systems (MCap and FP) was evaluated using Spearman’s rank correlation coefficients (*ρ*_s_). The strength of the correlation was classified as follows: |*ρ*_s_| ≤ 0.4, weak; 0.4 < |*ρ*_s_| ≤ 0.6, moderate; 0.6 < |*ρ*_s_| ≤ 0.8, strong; and |*ρ*_s_| > 0.8, very strong [41]. Additionally, the Bland–Altman analysis was performed to calculate the systematic bias and the limits of agreement (LoA, systematic bias ±1.96 standard deviations of the differences) for each jumping modality [42]. A single gold standard value (VJH_GSV_) was computed by averaging the estimates from the two gold standard systems—VJH_MCap_ calculated using Equation (1), and VJH_FP_ obtained via the NDI method—reflecting the high level of agreement observed between them.

The Friedman test was applied to identify significant differences between the VJH_GSV_ and the three IMU-based methods across the different jump modalities (DL-CMJ, R-CMJ, and L-CMJ). The effect size estimation was based on Kendall’s W coefficient of concordance [43]. Post hoc pairwise comparisons were conducted using the Conover test with Bonferroni correction to control for multiple comparisons. Bland–Altman analysis was also used to quantify the systematic bias and LoA between the VJH_GSV_ and each IMU method, as well as among the IMU methods themselves.

The test–retest reliability of the VJH estimates for each method was evaluated using the intraclass correlation coefficient with a two-way random-effects model (ICC(2,1)) and a 95% confidence interval (CI) [44]. The ICC(2,1) was chosen as an appropriate method to assess the test–retest reliability for scenarios where the same group of participants was tested multiple times, and both subjects and trials were treated as random effects [44,45]. This model accounts for the variance across subjects and measurements, making it suitable for assessing the consistency of repeated measurements across trials. ICC values were interpreted as: <0.5 poor reliability; 0.5 ≤ ICC < 0.75 moderate reliability; 0.75 ≤ ICC < 0.9 good reliability; and ICC ≥ 0.9 excellent reliability [44].

All statistical tests were performed with a significance level set at α = 0.05.

## 3. Results

### 3.1. Agreement Between Gold Standard Systems

**Figure 2:**
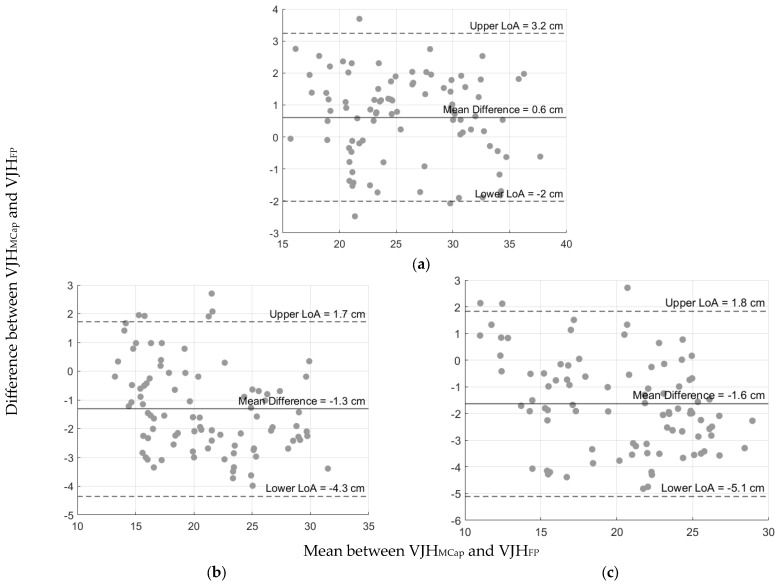
Bland–Altman Plots for VJH_MCap_ and VJH_FP._ (**a**) DL-CMJ; (**b**) R-CMJ; (**c**) L-CMJ. The center lines represent the systematic bias between systems and the upper and lower LoA [n = 90].

Table 1 reports the Spearman’s correlation coefficients, revealing significant and strong correlations between the two gold standard systems across all modalities (*ρ*_s_ ranging from 0.93 to 0.97, *p* < 0.001) and indicating a high level of agreement. Bland–Altman analysis (Table 1 and Figure 2) showed a systematic bias close to zero (0.6 cm) for the DL-CMJ and relatively narrow LoA (−2.0 to 3.2 cm, range = 5.2 cm). However, the R-CMJ and L-CMJ showed larger biases (−1.3 and −1.6 cm, respectively) and a wider LoA range (6.0 and 6.9 cm for the R-CMJ and L-CMJ, respectively), indicating greater variability in the estimates for these modalities. Nevertheless, due to the high agreement and the relatively low systematic bias between the MCap and FP, a single reference value (gold standard value, VJH_GSV_) was computed by averaging the estimates from MCap and FP and used for further comparisons with the IMU-based computation methods.

### 3.2. Agreement Between VJH_GSV_ and IMU Calculation Methods

For all jumping modalities, the IMU-based methods systematically underestimated the VJH compared to the VJH_GSV_ (Table 2). In particular,$\;{VJH}_{IMU}^{NDI}$ showed closer agreement with VJH_GSV_ across all modalities with respect to the FT and TOV methods, while ${VJH}_{IMU}^{FT}$ and ${VJH}_{IMU}^{TOV}$ exhibited larger deviations, especially in the R- and L-CMJ conditions. Nevertheless, the results from the Friedman test (Table 2) revealed significant differences among the methods for all the jumping conditions (*p*-values < 0.05).

The Bland–Altman analysis (Table 3) performed on all the computation methods revealed a systematic bias within 4.0 cm for the DL-CMJ across the different IMU-based calculation methods. However, the R-CMJ and L-CMJ conditions demonstrated larger systematic biases and wider LoA with respect to the DL-CMJ, particularly for the ${VJH}_{IMU}^{FT}$ (7.5 cm) and ${VJH}_{IMU}^{TOV}$ methods (7.4 cm), indicating reduced agreement with the VJH_GSV_ and declined SL-CMJ performances. The Conover post hoc test performed on the DL-CMJ revealed that both the NDI and the TOV methods significantly underestimated VJH compared to the VJH_GSV_ (Table 3, *p* < 0.001 after Bonferroni correction), while the FT method exhibited a non-significant difference (*p* = 0.080). The comparisons among the IMU methods showed no significant differences, with the FT and TOV methods yielding similar results (*p* = 1.000) in all jump modalities. In the case of the SL-CMJ, the NDI method showed no significant difference compared to the VJH_GSV_ for either the R-CMJ or the L-CMJ (*p* = 1.000 and *p* = 0.151, respectively), while the FT and TOV methods exhibited significant differences for both the R-CMJ and L-CMJ (*p* < 0.05).

Spearman’s rank correlation coefficients between the VJH_GSV_ and the IMU-based methods were calculated to evaluate their association in the different jump modalities.

The Spearman’s coefficients indicated a strong correlation between the VJH_GSV_ and the IMU-based methods (Table 4), with all correlations being significant (*p* < 0.05). Notably, the NDI method showed the highest correlation with the VJH_GSV_ (*ρ*_s_ ≥ 0.85 for all the jump modalities). Very strong correlations (*ρ*_s_ ≥ 0.89) were shown between all the IMU-based methods and the VJH_GSV_ in the DL-CMJ condition. However, the correlations were slightly lower for the R-CMJ and L-CMJ, particularly for the FT (*ρ*_s_ = 0.74 and *ρ*_s_ = 0.83, respectively) and TOV methods (*ρ*_s_ = 0.80 and *ρ*_s_ = 0.80, respectively), suggesting variability in the consistency of these methods across different jump modalities.

### 3.3. Test–Retest Reliability

The test–retest reliability of the VJH_GSV_ and of the three IMU-based calculation methods was assessed using the intraclass correlation coefficient (ICC) with a two-way mixed-effects model (ICC (2,1)). Table 5 provides the ICC values and the 95% CI for each method across the three different jump modalities.

The VJH_GSV_ showed excellent reliability across all modalities, with ICC values above 0.93 (Table 5), indicating high consistency in the VJH measurements from the gold standard methods between trials. Among the IMU-based methods, the NDI demonstrated the highest average ICC (0.88), suggesting strong reliability across all jump modalities. Specifically, the NDI method showed excellent reliability in the DL-CMJ (ICC = 0.92), and good reliability in the R-CMJ and L-CMJ (ICC = 0.85 and 0.86, respectively). The FT method exhibited moderate to high reliability depending on the jumping modality, with an average ICC of 0.83. The TOV method showed the lowest reliability among the IMU methods, with an average ICC of 0.79. Although it demonstrated good reliability in the DL-CMJ (ICC = 0.92), its reliability was substantially lower with respect to the other methods for the R-CMJ and L-CMJ (ICC = 0.72 and ICC = 0.73, respectively).

Excellent reliability was observed for the DL-CMJs across all the calculation methods, whereas the SL-CMJs generally showed lower reliability, possibly due to increased variability in single-leg jumps.

## 4. Discussion

VJH is recognized as a reliable metric for assessing CMJ performance [4,15,16]. The MCap systems and FPs are recognized as the gold standards for VJH assessment, but their substantial cost and lack of portability limit their use, particularly in ecological environments [17,18,19]. This study investigated whether a low-sampling-rate IMU could serve as a compact and feasible alternative to gold standard systems for estimating VJH in CMJs. The accuracy and reliability of three computational methods (NDI, TOV, and FT) for calculating VJH from a low-sampling-rate IMU was evaluated against the MCap and FP systems.

The results demonstrated that the MCap and FP systems showed strong agreement and minimal systematic bias, confirming their reliability as gold standards. These results are consistent with previous studies that have demonstrated the reliability of the MCap and FP systems for VJH measurements [20,22,25,33,39]. Furthermore, the present study confirmed that the NDI method is a robust approach to calculating VJH using dual FP systems, with minimal differences compared to the MCap systems [16,35,36]. Among the IMU-based methods, the NDI method yielded the highest accuracy, exhibiting the strongest correlation with the gold standards and the lowest systematic bias. This is consistent with earlier studies suggesting that NDI is the most accurate method for estimating VJH from IMUs [20]. However, the present study extends these findings by demonstrating that even at a low sampling rate, NDI retains strong performance, which is notable given the typical reliance on high-frequency data in previous research [16,25].

A systematic bias of approximately 4.0 cm was observed when comparing the NDI method to the gold standard systems for DL-CMJs. This bias could be attributed to differences in how the COM is approximated across systems. The IMU, positioned at the L5 vertebra, estimated the COM based on a single point [39], whereas the MCap system utilized multiple markers on the pelvis, potentially introducing discrepancies, as identified in the Bland–Altman analysis (Table 3). Similarly, it is important to note that FPs estimate COM displacement based on ground reaction forces, which may also lead to divergent results. For the single-leg CMJs (SL-CMJs), the bias was more pronounced due to the increased difficulty in estimating the COM in a unilateral stance. This could be improved by directly placing a marker on the IMU sensor, as suggested by [39]. Despite these challenges, the NDI method demonstrated a high level of agreement and reliability with the gold standards, corroborating findings from prior research [20,39]. A notable finding in this study was the strong correlation between the IMU-based NDI method and the gold standard systems, despite the observed systematic bias. This suggests that calibration equations could be developed to reduce measurement errors and align IMU-based estimates more closely with gold standard outputs.

In the DL-CMJs, the FT and TOV methods performed comparably to the NDI method, exhibiting similar systematic bias and correlation coefficients relative to the gold standards. However, their performance declined significantly in the SL-CMJs; this was likely due to the increased difficulty in maintaining consistent posture at takeoff and landing, which is a critical assumption for the FT method [20,39]. Additionally, the identification of these events is even more critical when considering the relatively low sampling frequency of the IMU compared to the FP and MCap systems, as well as that of the IMUs used in other similar studies [16]. Lastly, the identification of these events is particularly sensitive to sensor noise and soft tissue artifacts, which can introduce errors, as reported in previous studies [47]. While the NDI method is also affected by these issues, its sensitivity to such disturbances is generally lower [20]. Similar limitations have been noted in studies using IMUs with a higher sampling rate, but our study suggests that even at reduced sampling rates, these effects can still influence performance, warranting further research [16,25]. Nevertheless, advanced strategies to reduce these fluctuations should be investigated to potentially improve the precision of IMU-based methods for estimating VJH.

An important implication of the present study is related to its obtainment of reliable and accurate VJH estimates using a low-sampling-rate IMU, which is advantageous for applications requiring low power consumption, portability, and fast data processing, such as in-field performance testing, rehabilitation monitoring, and sports analytics. This is particularly relevant for industries exploring the integration of IMUs with TinyML (machine learning on microcontrollers) and edge computing, where real-time analysis and long duration batteries are crucial. Our findings suggest that the NDI method could be integrated into such platforms to provide reliable VJH estimates during sport practice, offering a feasible alternative to traditional systems that are less adaptable to real-world, ecological environments.

### Limitations and Future Developments

Despite the promising findings of this study, several limitations should be acknowledged, along with considerations for future developments.

One of the main limitations of the present study is the relatively small and homogeneous sample size, which is sufficient for statistical power analysis, but may limit the generalizability of the findings. Although the sample size included both men and women to introduce heterogeneity and reflect real-world differences, future studies should consider incorporating a larger and more diverse sample size to enable subgroup analysis and to enhance the generalizability of the results. This approach will allow researchers to provide robust validation of the NDI method to estimate VJH across populations with varying physical characteristics and performance levels.

Moreover, this study utilized an FP with a sampling frequency of 200 Hz, which, while it was the maximum feasible within the technical limitations of our laboratory, does not represent the gold standard. We acknowledge that a higher sampling frequency could potentially yield more precise reference data. However, given these constraints, our results provide a feasible alternative for settings where access to high-end equipment is limited. Future research should employ higher sampling frequencies to validate and refine the findings of this study.

The NDI method demonstrated promising results in estimating VJH using low-sampling-rate IMUs. However, systematic errors that could be attributed to soft tissue artifacts, variations in IMU placement, and the inherent limitations of low-sampling-rate systems were observed, particularly in the SL-CMJs. For this reason, future studies should focus on the development and validation of calibration equations to address these systematic biases and improve the precision and reliability of VJH estimates from IMU-based data.

Lastly, the present study focused primarily on CMJs performed under controlled laboratory conditions. Different jump modalities and less controlled environments (e.g., on-field conditions) could impact the performance of IMU-based methods. Expanding this analysis to other jump types, such as squat jumps and drop jumps, is crucial for broadening the applicability of the proposed methods. Moreover, testing under ecological conditions representative of actual sports scenarios, such as volleyball, basketball, or football, will provide valuable insights into the robustness and versatility of these approaches.

## 5. Conclusions

This study demonstrates that the NDI method offers the highest accuracy and agreement with gold standard systems for estimating VJH from low-sampling-frequency IMUs. The findings confirm that while the TOV and FT methods are suitable for DL-CMJs, their accuracy diminishes significantly in SL-CMJs. This reduction in performance is likely attributable to inherent challenges in accurately measuring takeoff velocity and flight time, particularly under asymmetric conditions.

The results emphasize the potential of IMU sensors as a viable and practical alternative to traditional MCap and FP systems. This approach is especially beneficial when portability, ease of use, and cost-effectiveness are key factors to be considered. Although some limitations remain, such as the need for a larger, more diverse sample size and for a calibration equation to mitigate systematic errors, this study lays the groundwork for future advancements in wearable sensor technologies. By addressing these limitations, IMU-based methods will enable broader adoption across clinical, athletic, and recreational settings, offering accessible solutions for the monitoring and enhancing of vertical jump performance.

## Figures and Tables

**Table 1:** Median (IQR), Spearman’s coefficients (*ρ*_s_), and systematic bias with LoA from gold standard methods.

Modality	VJH_MCap_ (cm)	VJH_FP_ (cm)	*ρ*_s_	Systematic Bias (LoA) (cm)
DL-CMJ	26.2 (5.4)	25.6 (5.5)	0.97 *	0.6 (−2.0 to 3.2)
R-CMJ	20.2 (4.7)	21.5 (5.2)	0.94 *	−1.3 (−4.3 to 1.7)
L-CMJ	19.5 (4.4)	21.1 (5.0)	0.93 *	−1.6 (−5.1 to 1.8)

* Significant correlation (*p* < 0.001). DL-CMJ = double-leg countermovement jump; R- = right; L- = left; VJH = vertical jump height; MCap = motion capture systems; FP = force platform; *ρ*_s_ = Spearman’s rank correlation coefficient; LoA = level of agreement.

**Table 2:** Median (IQR) for the VJH_GSV_ and the three IMU-based VJH computations: NDI, FT, and TOV and different jumping modalities. The two columns on the right-hand side display the Friedman test results in terms of χ^2^ and Kendall’s W.

Modality	VJH_GSV_ (cm)	${VJH}_{IMU}^{NDI}$(cm)	${VJH}_{IMU}^{FT}$(cm)	${VJH}_{IMU}^{TOV}$(cm)	χ^2^(3)	W
DL-CMJ	24.6 (9.0)	21.1 (6.8)	21.1 (9.0)	21.2 (9.1)	23.87 *	0.44
R-CMJ	20.0 (8.6)	19.1 (7.5)	13.3 (5.9)	13.3 (6.7)	44.53 *	0.83
L-CMJ	21.6 (7.6)	19.0 (7.8)	13.8 (5.6)	13.9 (8.1)	49.20 *	0.91

* Significant *p*-values (*p* < 0.05); DL-CMJ = double-leg countermovement jump; R- = right; L- = left; VJH = vertical jump height; GSV = gold standard values; IMU = inertial measurement unit; NDI = numerical double integration; FT = flight time; TOV = takeoff velocity.

**Table 3:** Systematic bias, LoA, and *p*-values after Bonferroni correction obtained from the Bland–Altman analysis and Conover test.

Comparison	Modality	Systematic Bias (LoA) (cm)	p_bonf_
VJH_GSV_ vs. ${VJH}_{IMU}^{NDI}$	DL-CMJ	4.0 (−0.9 to 8.8)	<0.001 *
	R-CMJ	1.5 (−3.9 to 6.9)	1.000
	L-CMJ	1.9 (−3.2 to 7.1)	0.151
VJH_GSV_ vs. ${VJH}_{IMU}^{FT}$	DL-CMJ	3.0 (−0.3 to 6.4)	0.080
	R-CMJ	7.8 (−0.3 to 15.9)	<0.001 *
	L-CMJ	7.1 (0.4 to 13.8)	<0.001 *
VJH_GSV_ vs. ${VJH}_{IMU}^{TOV}$	DL-CMJ	3.4 (−3.7 to 10.4)	0.004 *
	R-CMJ	7.7 (1.2 to 14.1)	<0.001 *
	L-CMJ	7.1 (0.5 to 13.7)	<0.001 *
${VJH}_{IMU}^{NDI}vs.{VJH}_{IMU}^{FT}$	DL-CMJ	−0.9 (−3.9 to 2.1)	0.273
	R-CMJ	6.3 (−2.6 to 15.2)	0.002 *
	L-CMJ	5.1 −2.8 to 13.1)	0.020 *
${VJH}_{IMU}^{NDI}vs.{VJH}_{IMU}^{TOV}$	DL-CMJ	−0.6 (−9.2 to 8.1)	1.000
	R-CMJ	6.2 (−0.1 to 12.4)	<0.001 *
	L-CMJ	5.2 (−0.9 to 11.2)	0.002 *
${VJH}_{IMU}^{FT}vs.{VJH}_{IMU}^{TOV}$	DL-CMJ	0.4 (−6.8 to 7.5)	1.000
	R-CMJ	−0.1 (−9.3 to 9.1)	1.000
	L-CMJ	0.1 (−8.5 to 8.6)	1.000

* Significant *p*-values (*p* < 0.05). DL-CMJ = double-leg countermovement jump; R- = right; L- = left; VJH = vertical jump height; GSV = gold standard value; IMU = inertial measurement unit; NDI = numerical double integration; FT = flight time; TOV = takeoff velocity.

**Table 4:** Spearman’s rank correlation coefficients between VJH_GSV_ and IMU-based methods.

Method	Modality	VJH_GSV_	${VJH}_{IMU}^{NDI}$	${VJH}_{IMU}^{FT}$	${VJH}_{IMU}^{TOV}$
VJH_GSV_	DL-CMJ	-			
	R-CMJ	-			
	L-CMJ	-			
${VJH}_{IMU}^{NDI}$	DL-CMJ	0.90 *	-		
	R-CMJ	0.85 *	-		
	L-CMJ	0.85 *	-		
${VJH}_{IMU}^{FT}$	DL-CMJ	0.93 *	0.95 *	-	
	R-CMJ	0.74 *	0.63 *	-	
	L-CMJ	0.83 *	0.68 *	-	
${VJH}_{IMU}^{TOV}$	DL-CMJ	0.89 *	0.83 *	0.89 *	-
	R-CMJ	0.80 *	0.77 *	0.61 *	-
	L-CMJ	0.80 *	0.84 *	0.67 *	-

* Significant correlation (*p* < 0.05). DL-CMJ = double-leg countermovement jump; R- = right; L- = left; VJH = vertical jump height; GSV = gold standard values; IMU = inertial measurement unit; NDI = numerical double integration; FT = flight time; TOV = takeoff velocity.

**Table 5:** Intraclass correlation coefficients ICC(2,1) with 95% CI of the calculation methods.

	Modality	VJH_GSV_	${VJH}_{IMU}^{NDI}$	${VJH}_{IMU}^{FT}$	${VJH}_{IMU}^{TOV}$
ICC(2,1) (95% CI)	DL-CMJ	0.96 (0.92–0.98)	0.92 (0.85–0.97)	0.94 (0.88–0.97)	0.92 (0.85–0.96)
	R-CMJ	0.93 (0.89–0.97)	0.85 (0.73–0.93)	0.74 (0.58–0.88)	0.72 (0.55–0.86)
	L-CMJ	0.93 (0.86–0.97)	0.86 (0.74–0.93)	0.90 (0.82–0.96)	0.73 (0.56–0.87)
	Average	0.94 (0.89–0.97)	0.88 (0.77–0.94)	0.83 (0.76–0.94)	0.79 (0.65–0.90)

Eighteen participants and 5 measurements. ICC type as referenced by [46].

## Data Availability

The raw data supporting the conclusions of this article will be made available by the authors upon request.
